# Identification of HSP90 as Potential Biomarker of Biliary Atresia Using Two-Dimensional Electrophoresis and Mass Spectrometry

**DOI:** 10.1371/journal.pone.0068602

**Published:** 2013-07-11

**Authors:** Rui Dong, Panmo Deng, Yanlei Huang, Chun Shen, Ping Xue, Shan Zheng

**Affiliations:** Department of Pediatric Surgery, Children’s Hospital of Fudan University, and Key Laboratory of Neonatal Disease, Ministry of Health, Shanghai, China; The University of Hong Kong, Hong Kong

## Abstract

Biliary atresia (BA) is a devastating cholestatic liver disease targeting infants. Current diagnosis depends on surgical exploration of the biliary tree. The aim of the present study was to identify potential biomarkers for the diagnosis of biliary atresia (BA). Two-dimensional electrophoresis was utilized for the identification of proteins that were differentially expressed in liver biopsies of 20 BA patients and 12 infants with non-BA neonatal cholestasis (NC) as controls. Using mass spectrometry, we identified 15 proteins with expressions significantly altered. Out of the 15 proteins identified, heat shock protein (HSP) 90 was the most significantly altered and was down-regulated in BA samples compared to NC samples using immunoblotting analysis. Our findings suggest that HSP90 might be a potential biomarker for the diagnosis of BA and may be used for monitoring further development and therapy for BA. This study demonstrated that a comprehensive strategy of proteomic identification combined with further validation should be adopted in biomarker discovery.

## Introduction

Biliary atresia (BA) is a devastating cholestatic liver disease affecting neonatal infants. BA occurs in 1/15,000 children and is more common in females than males. The etiology of BA is unknown and treatments are only partially successful [1], [2], [3]. Early detection of BA and surgical intervention using the Kasai procedure is correlated with satisfactory long-term outcomes [4], [5]. Currently, there is an urgent need to devise tools for the early diagnosis and progression of BA. Among these tools, validated biomarkers are viewed as the most important.

Proteomic analyses, a powerful tool for the global evaluation of protein expression, has been widely applied in the analysis of a number of diseases [6], [7], [8], [9]. Biomarker discovery and validation is a central application in current proteomic research to improve the diagnosis, treatment monitoring and prognosis of many types of disease [6], [7], [8], [9]. Associations between protein alterations and disease processes-analyses of the proteome-could be more informative and advantageous than genomics or transcriptomics alone, since there are also factors related to molecular changes in translation, post-translational modification and intracellular mislocalization involved in disease initiation and growth [10]. Two-dimensional gel electrophoresis (2-DE) is an established technology for the separation of proteins. Despite a number of limitations, 2-DE gel resolution is impressive, rendering this technology a preferred tool in many proteomics studies [11], [12], [13]. Quantitative proteomics based on 2-DE coupled with peptide mass fingerprinting remains one of the most widely used quantitative proteomic approaches in many research studies, and has successfully identified novel proteins [14], [15], [16]. Plasma/serum peptides or proteins that are biological indicators are known as biomarkers. However, there is difference between tissues especially with tissue specific diseases. Therefore, in the present study we proposed experiments aimed to detect differences in protein expression in liver biopsy samples from infants with BA and non-BA neonatal cholestasis infants and evaluate the identified proteins for the potential to be utilized as biomarkers. Alterations in protein expression between BA and non-BA neonatal cholestasis liver biopsies were evaluated by 2-DE and MALDI-TOF-MS.

## Methods

### Patients

The study group consisted of 20 infants with BA and 12 infants with non-BA neonatal cholestasis infants (NC). Infants diagnosed with BA or NC were included in the study based on clinical pattern, cholangiogram, and histological findings. Study participants were prospectively recruited from the Children’s Hospital of Fudan University. Clinical data for BA and NC infants were obtained retrospectively using the clinical files (Table 1). Entry criteria for the BA study included type III classification of the BA phenotype, age younger than 90 days, and serum direct or conjugated bilirubin levels >20% of total and >2 mg/dL. Children with liver failure, malignancy, hypoxia, shock, ischemic hepatopathy within the preceding 2 weeks, extracorporeal membrane oxygenation–associated cholestasis, and/or prior hepatobiliary surgery were excluded from the present study. Children with primary hemolytic disease, drug or total parenteral nutrition–associated cholestasis, bacterial or fungal sepsis, or birth weight <1500 g were also excluded from the present study unless they were definitively diagnosed as having BA or another cholestatic disease. The 12 infants with neonatal cholestasis with causes other than BA presenting during the same time frame were used as controls, called the NC group. A retrospective review of case and imaging records of these infants identified the underlying etiology in these cases, which included 10 neonatal hepatitis, 1 Alagille syndrome, and 1 biliary hypoplasia. The Ethics Committee at the Children’s Hospital of Fudan University approved all studies. Voluntary informed written consent was obtained from the parents of all participants prior to enrollment in this study.

**Table:** Table 1. Distribution of study subjects and liver function tests.

	BA	NC	P
Age (days)*	71.50±17.52	65.91±12.33	0.25
Male/Female	12/8	7/5	0.31
Diagnosis type	III^1^		N/A
TB (μmol/L)	198.1±105.45	161.33±52.79	0.61
DB (μmol/L)	144.52±74.38	121.92±37.67	0.47
DB/TB	0.74±0.59	0.76±0.51	0.09
AST (IU/L)	262.5±237.50	230.9±208.43	0.18
ALT (IU/L)	121.1±113.75	142.3±147.97	0.45
γ-GGT	800.88±727	253.0±215.64	0.01

1. Type III biliary atresia refers to the discontinuity of both right and left hepatic ducts to the level of the porta hepatis. Unfortunately, type III BA is common, accounting for >90% of cases. *at liver biopsy sample day.

BA: biliary atresia; NC: non-BA neonatal cholestasis infants.

ALT: Alanine transaminase; AST: Aspartate transaminase; DB: Direct bilirubin; TB: Total bilirubin; γ-GGT: Gamma glutamyl transpeptidase.

### Sample Collection and Preparation

Liver biopsies were immediately snap-frozen in liquid nitrogen and stored at −80°C until analyses. Proteins were extracted from human liver biopsy tissue as described [17], [18]. Briefly, liver tissue (100 mg) was homogenized in 2 mL buffer (50 mM Tris-HCl, pH 7.2) containing 1 mM PMSF using a homogenizer. The mixture was centrifuged at 1,000 g for 5 min to remove debris. Then, the homogenate was centrifuged for 30 min at 4^°^C. The supernatant was taken as the soluble fraction and TCA (50% w/v) was added to a final concentration of 10% w/v (containing 20 mM DTT); the solution was then allowed to stand on ice for 30 min. The protein precipitate was collected and centrifuged in a microcentrifuge for 10 min at 4^°^C and was washed in triplicate in 10% TCA (containing 20 mM DTT). The precipitate was washed twice in acetone (containing 20 mM DTT) and dried using an air stream. The dry pellet was dissolved with a vortex in a lysis solution (7 mol/L urea, 2 mol/L thiourea, 2% w/v CHAPS, 2% w/v SB3−10 (N-decyl-N-N’-dimethyl−3-ammonio−1-propane sulfonate), 40 mM Tris, 2 mM TBP (tributylphosphane), 0.2% w/v Bio-Lyte pH 3–10, 1050 U/mL DNase, and 25 μg/mL RNase) and allowed to stand for 1 h at room temperature. Following centrifugation for 10 min at 15°C, the supernatant was used as the two-dimensional polyacrylamide gel electrophoresis (2-DE) sample for the soluble fraction. The protein samples were stored in aliquots at −80°C until further use. Protein concentration of 2-DE samples was determined using a Bradford Protein Assay Kit (Bio-Rad, Richmond, CA).

### Two-Dimensional Gel Electrophoresis

First-dimensional gel separation was performed with 17 cm IPG strips (pH 3–10, pH 4–7, pH 5–8) following the manufacturers’ protocol with minor modifications. Samples containing up to 100 μg protein were diluted with 320 μL of rehydration solution (7 mol/L urea, 2 mol/L thiourea, 2% CHAPS, 100 mM DTT, 0.2% w/v Bio-Lyte pH 3–10, and trace bromophenol blue), and applied to strips for overnight rehydration at 50 V. Proteins were focused for 1 h at 150 V, 1 h at 250 V, 1 h at 1000 V. A gradient was then applied from 1000 to 10,000 V in 5 h, and focusing was continued at 10,000 V for 6 h to give a total of 88 kVh on a Protean IEF Cell. Following IEF, strips were equilibrated for 15 min in 6 mol/L urea, 2% SDS, 0.05 mol/L Tris-HCl, pH 8.8, and 30% glycerol containing 2% DTT, and then equilibrated again for 15 min in the same buffer containing 2.5% iodoacetamide. Equilibrated IPG strips were transferred onto 12% uniform polyacrylamide gels and then were run on the Protean Plus Dodeca Cell (Bio-Rad, USA) at 24 mA per gel for 5 h and 30 min. The gels were visualized using silver staining methods as previously shown [19]. After staining, 2D gels were scanned using a Fluor-STM Multimager (Bio-Rad, USA) and images were analyzed using PDQuest 7.1.1 (Bio-Rad, Hercules, USA).

### Image Analysis

Image analysis was performed using PDQuest 2D-analysis software (Bio-Rad, CA). Spot intensity was quantified automatically by calculation of spot volume following normalization of the image by taking the ratio of intensity of one spot to total spots, and expressed as a fractional intensity. Only those spots with 3.5-fold (t test, *p*<0.05) or more changes in expression intensity were selected for further MALDI-TOF-MS (Shimadzu/Kratos, Manchester, UK) analyses.

### In-Gel Digestion of Proteins

Protein spots of interest were cut manually from the gel and placed into siliconized microcentrifuge tubes. Gel fragments were destained in solution that consisted of 100 mM Na_2_S_2_O_3_ and 30 mM K3Fe (CN) 6 (V/V, 1: 1) and dehydrated by washing twice for 10–15 min with 25 mM NH_4_HCO_3_ in 50% acetonitrile until shrunken and white. The protein-containing gel-spots were reduced in the reduction buffer (25 mM NH_4_HCO_3_ and 10 mM DTT) for 1 h at 56°C and then alkylated in the alkylation buffer (25 mM NH_4_HCO_3_ and 55 mM iodoacetamide) in the dark for 30 min at room temperature. Gel pieces were washed twice with 100 mM NH_4_HCO_3_ and dehydrated using 100% acetonitrile. Ten microliters modified trypsin (8 μg/mL) (Sigma-Aldrich, St. Louis, MO) was added to each tube. Tubes were sealed with parafilm and digestion was performed for 16 h at 30°C. When digestion was completed, 100 μL of 100 mM NH_4_HCO_3_ was added and tubes were sonicated for 10 min. The supernatant was removed from each tube and transferred into new tubes. Further recovery of the peptides was accomplished by two extractions with 50% acetonitrile/5% TFA. All supernatants were pooled and concentrated and desalted by ZipTips (Millipore, Bedford, MA, USA) prior to application to the sample plate.

### MALDI-TOF-MS Spectra Analysis of the HSP90

MALDI-TOF-MS analysis of the samples was performed using a mass spectrometer Autoflex (Bruker Daltonics, Billerica, MA) in a positive ion reflector mode. The ion acceleration voltage was 20 kV. Each spectrum was internally calibrated with the masses of two trypsin autolysis products (porcine and bovine trypsin). For PMF identification, tryptic peptide mass maps were transferred with the MS BioToolsTM program (Bruker Daltonics) using MASCOT software (version 2.3) (Matrix Science). The National Center for Biotechnology non-redundant (NCBInr release 20091112, containing 10,032,801 sequences) database was then searched with human as the taxonomy. Up to one missed tryptic cleavage was considered and a mass accuracy of 100 ppm was used for all the tryptic-mass searches.

### Identification of HSP90 by LC-MS/MS

In the digestion solution of each concentrated fraction, the analysis of each candidate protein biomarker was performed by a standard protocol. Briefly, each fraction was dissolved in 25 mM NH4HCO3, and reduced with 10 mM DTT for 1 hour, alkylated by 40 mM iodoacetamide in the dark for 45 min at room temperature; then 40 mM DTT was added to quench the iodoacetamide for 30 min at room temperature. Then, proteins were lysed with 20 ng of modified trypsin (Promega Corporation, Madison, WI) in 25 mM NH4HCO3 overnight at 37°C. The supernatant was collected and peptides were further extracted in 0.1% acetic acid and 60% acetonitrile. Peptide extracts were vacuum dried and resuspended in 20 μl of water for mass analysis. Protein digests obtained above were loaded onto a C18 column (100 mm × 100 μm) packed with Sunchron packing material (SP-120-3-ODS-A, 3 μm) and followed with nano-LC-ESI-MS/MS analysis. The LTQ mass spectrometer was operated in a data-dependent mode, in which the initial MS scan recorded the m/z ratios of ions over the mass range from 400–2000 Da, and then the five most abundant ions were automatically selected for subsequent collision-activated dissociation. All MS/MS data were searched against a human protein database downloaded from NCBI using the SEQUEST program (Thermo, USA).

### Immunoblotting Analysis for HSP90

Tissue samples were lysed in radioimmuno-precipitation assay (RIPA) lysis buffer, and phenylmethylsulfonyl fluoride (PMSF) (1 mM, final concentration) was added after several minutes. Protein samples were electrophoresed on 10% denaturing sodium dodecyl sulfate gels and transferred to a polyvinylidene difluoride (PVDF) membrane. The membrane was incubated with an HSP90 antibody (R&D Systems, Minneapolis, MN, USA) followed by washing and incubation with an HRP-goat anti-rabbit antibody (Sigma-Aldrich, St. Louis, MO, USA). The membrane was stripped and incubated with a mouse anti-β-actin antibody (Sigma-Aldrich, St. Louis, MO, USA) followed by incubation with an HRP-goat anti-mouse antibody (Kangcheng Co., Ltd. Shanghai, China). HSP90 and β-actin bands were visualized using enhanced chemiluminescence (ECL) detection (Amersham Pharmacia Biotech Co., Ltd. UK) and analyzed with gray scale scanning quantitation.

### Statistical Analyses

Data are expressed as mean ± standard error of the mean (SEM). A Student’s t-test was used to determine statistically significant differences between groups. *p*-values <0.05 were considered statistically significant. Standard clinical parameters (Table 1) were evaluated using a Mann-Whitney rank sum or t test. All analyses were performed using the SPSS Version 13.0 software.

## Results

### Differential Protein Expression in BA Patients

**Figure 1:**
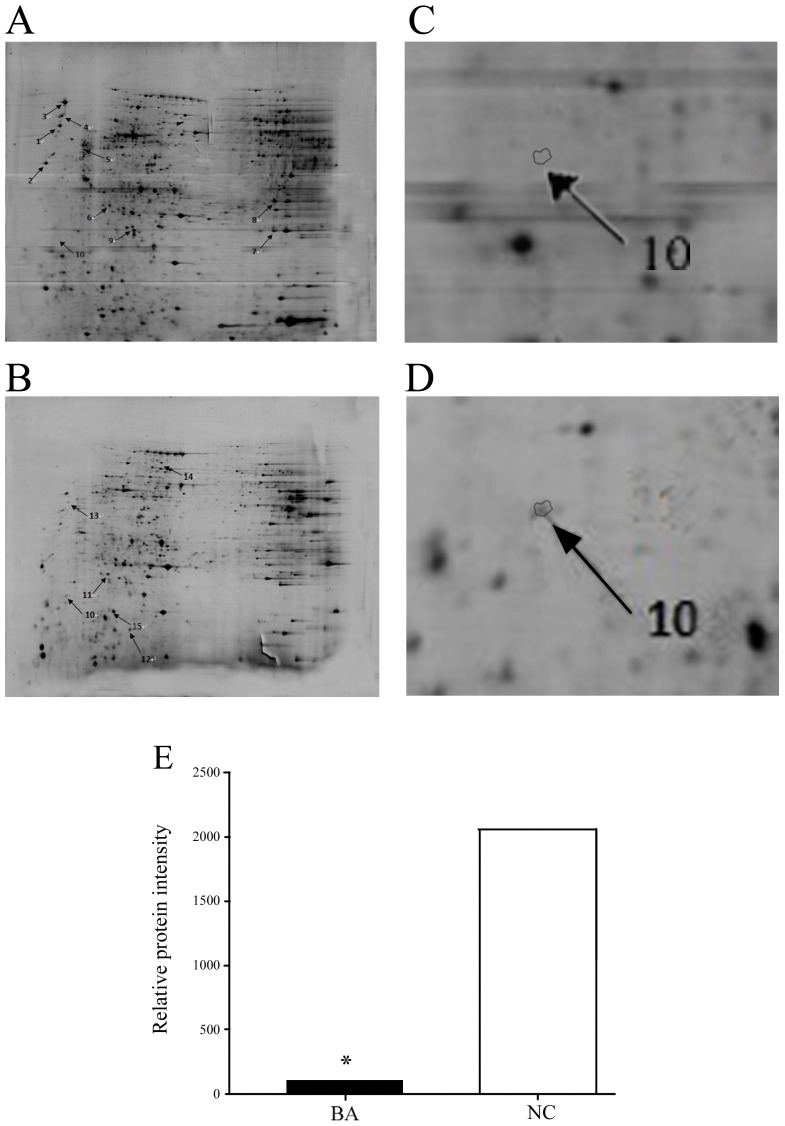
Representative 2-DE gel images. Identical images are shown in A and B. (A) Highlighted spots are more abundant in BA than NC controls. (B) Highlighted spots are more abundant in NC than BA patients. Numbers beside the spots correspond to Table 2. (C) and (D) Representative Spot 10, cropped 2-DE gel images of HSP90 in BA patients and NC controls. (E) HSP90 showed the most significant differences in expression between BA (101.0±2.3) and NC controls (2060.0±8.6) (*p*<0.0001). HSP90 was down-regulated more than 20-fold in BA patients when compared to NC controls. BA: biliary atresia; NC: non-BA neonatal cholestasis infants.

**Figure 2:**
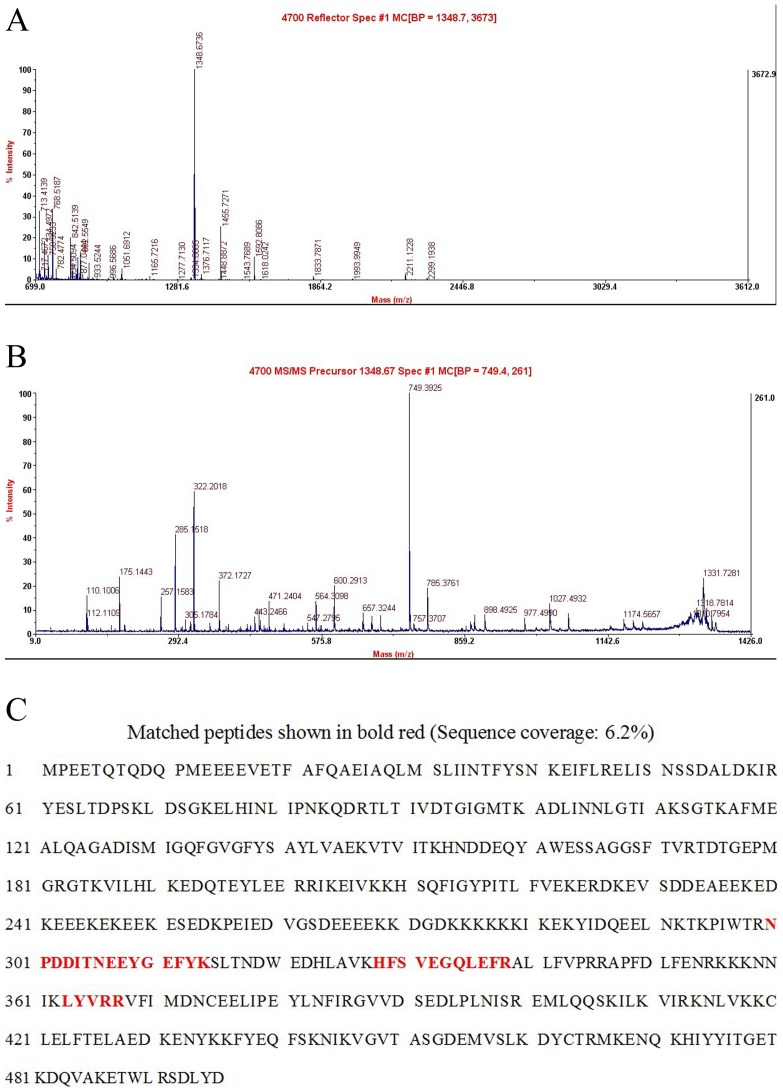
A: MALDI/Mass spectrum of the protein spot of HSP90 identified using MALDI-TOF-MS. B: MALDI-TOF-TOF mass spectrum of the mixture of tryptic peptides derived from Spot 10 (HSP90); C: Matching of protein Spot 10 (HSP90) Peptide Mass Fingerprint data in database.

Protein expression profiles in BA and NC liver biopsies were obtained using 2-DE gel analyses. Gel images and representative 2-DE maps were unambiguously matched using the PD-Quest software, and displayed well-resolved and reproducible profiles for both BA and NC controls. Approximately 800–1000 protein spots were detected with silver staining in a single 2-DE gel. The quantity of each spot in a gel was normalized as a percentage of the total quantity of all spots in the gel. In comparison with 2-DE patterns, differentially expressed proteins were defined as statistically significant on the basis of a 3.5-fold up-regulation and 3.5-fold down-regulation in BA patients compared with NC controls, or more changes in expression intensity (*p*<0.05). According to these criteria, 18 spots were selected and analyzed using MALDI-TOF-MS. Fifteen proteins from the 18 spots were identified (Table 2). HSP90 was up-regulated more than 20-fold in NC when compared with BA patients (Figure 1). The MALDI/Mass spectrum of the protein spot is shown in Figure 2. Redundancy of proteins appearing in the database under different names and accession numbers was removed. If more than one protein was identified in one spot, the single protein member with the highest protein score (top rank) was singled out from the multiprotein family.

**Table 2:** Differentially expressed proteins identified by MALDI-TOF-MS in liver biopsy samples from BA and NC patients.

Spot Number	Protein name	MW (Da)	pI	Fold change (BA/NC)	Score
1	Calreticulin precursor variant	46890.1	4.3	629.6	100
2	Calreticulin precursor	48111.8	4.29	359	100
3	Chain C, crystal structure of the globular domain ofhuman calreticulin	30092.6	4.74	230	100
4	Thyroid hormone binding protein precursor	57068.7	4.82	215	100
5	Vimentin	41537	4.82	26.1	100
6	Chain A, crystal structure of truncated humanrhogdi triple mutant	20588.5	5.36	6.9	96.55
7	Mn-superoxiddismutase	19345.8	7.25	4.7	99.95
8	Albumin, isoform CRA_o	29228.8	6.97	4.7	100
9	Myosin, light polypeptide 6, alkali, smooth muscle andnon-muscle, isoform CRA_d	13150.2	4.47	3.4	99.82
10	Heat shock protein 90kDa (cytosolic), class A member 1,isoform CRA_b	57697.1	4.92	0.05	100
11	Chain A, solution structure of Bb’ domains of humanprotein disulfide isomerase	25518.1	4.84	0.04	100
12	Annexin A6, isoform CRA_c	75229.2	5.46	0.01	100
13	Unnamed protein product	38608.2	5.19	0.036	98.38
14	Carbamoylphosphate synthetase	55696.2	8.95	0.026	100
15	Chain A, human protein disulfide isomerase, Nmr, 40 structures	13248.7	5.94	0.006	100

BA: biliary atresia; NC: non-BA neonatal cholestasis infants.

### Validation of the Significantly Differentially-Expressed Protein HSP90 using Immunoblotting Analysis

**Figure 3:**
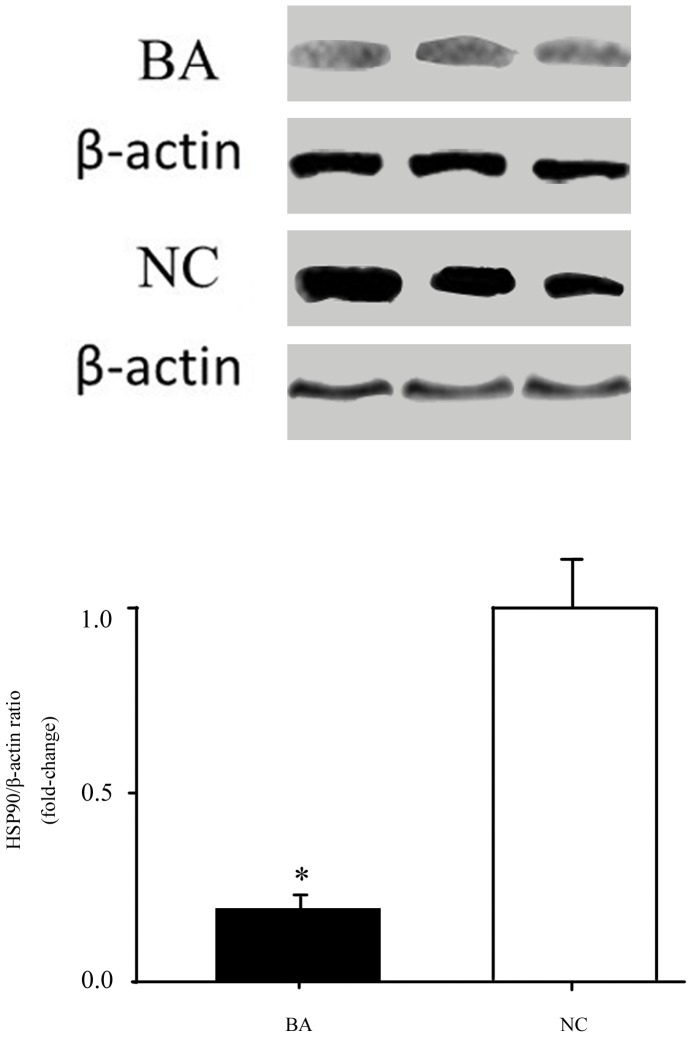
Immunoblotting analysis of HSP90 protein expression. HSP90 protein expression (HSP90/β-actin ratio) was significantly lower in BA infants (53279±3408) than NC controls (276669±19421) (*p* = 0.03). BA: biliary atresia; NC: non-BA neonatal cholestasis infants.

To evaluate HSP90 protein expression in liver biopsy tissues, we assessed HSP90 protein levels in BA infants and NC controls. Protein expression in liver biopsy tissues was evaluated using immunoblots and NC liver biopsy tissue samples as controls. The average HSP90 expression level (HSP90/β-actin ratio) was significantly decreased in BA infants (53279±3408) compared to NC controls (276669±19421) (*p* = 0.03) (Figure 3), which was correlated with the 2-DE results.

## Discussion

Biliary atresia (BA) is a destructive inflammatory obliterative cholangiopathy affecting both intrahepatic and extrahepatic bile ducts in neonates. The progression of obstructive biliary diseases is associated with proteomic changes, which may be partially due to changes in the blood and tissues composition of patients. For instance, LPS-binding protein mediates LPS-induced liver injury and mortality during biliary obstruction [20]. Proteins are sometimes secreted by cholangiocytes or hepatocytes in response to cholestatic diseases such as BA [21], [22], [23]. Cholangiocytes are the target cells of a number of diseases termed cholangiopathies, including BA [24]. Cholangiocytes line the intra-and extra-hepatic bile ducts of the biliary system, which is comprised of a series of interconnected tube-like structures that drain bile from the liver and deliver it to the gallbladder or duodenum [24]. Cholangiocytes modify the composition of bile that is secreted at the canalicular membranes of hepatocytes as it flows through the biliary system [25]. This modification involves the secretion and absorption of water, electrolytes and other organic solutes from hepatocellular bile [26]. Although the etiology of bile ducts injury in BA in unknown, it is postulated that a pre- or perinatal viral infection initiates cholangiocyte apoptosis and release of antigens that leads to further bile duct injury [27]. When the biliary tract suffers from infection, cholangiocytes secrete vascular endothelial growth factor [28] and serotonin [29], which may contribute to the process of BA [30].

In the present study we compared global protein profiles between liver biopsy tissues of BA patients and NC controls using a 2-DE and MALDI-TOF-MS approach. The goal of our study was to compare BA and NC tissue protein expression to reveal potential proteins that play a key role in the pathogenesis of BA and that might function as biomarkers. A total of 15 differentially expressed proteins were identified, most of which were involved in specific biological process including cell transformation, protein folding, cell proliferation and apoptosis. Of the 15 identified proteins, nine were up-regulated and six were down-regulated in the liver biopsy tissues of BA patients. HSP90 showed the most remarkable changes between BA and NC patients; the mass spectrometric identification of HSP90 confirmed these results. Significant elevations in HSP90 from the liver biopsy tissues of BA patients were also validated using immunoblotting analysis. Indeed, the results showed that the mean level of HSP90 in BA patients was significantly lower than in NC controls, which was correlated with the 2-DE results.

Heat shock proteins (HSP) are a class of functionally related proteins involved in the folding and unfolding of other proteins. HSP expression is increased when cells are exposed to elevated temperatures or other stress [31]. This increase in expression is transcriptionally regulated. The marked upregulation of HSPs is a key part of the heat shock response and is induced primarily by heat shock factor (HSF) [32]. Heat-shock proteins are named according to their molecular weight. Some HSP have been identified to play a role in BA; for example, the collagen chaperone heat shock protein 47 has been shown to be involved in the development of fibrosis in BA [33], [34]. The most widely-studied HSPs are Hsp90 that are specialized molecular chaperones that fulfill the folding, maintenance of structural integrity and conformational regulation of a subset of proteins involved in important cellular processes, such as transduction of signals and cell cycle control. Hsp90 has also been shown to suppress the aggregation of a wide range of “client” or “substrate” proteins and acts as a general protective chaperone [35], [36], [37]. Indeed, HSP90 proteins regulate apoptosis by inhibiting calcium-dependent proteases, calpains, and protect human neuroblastoma cells from hypoxia/reoxygenation-induced apoptosis requiring calpains [38], [39]. HSP90 is also a major contributing protein of protection against 3-hydroxykynurenine-induced neuronal apoptosis [40], and has a protective reaction during acute toxic stress [41]. Hsp90 plays a significant role in the growth and development of organisms carrying out conformational regulation of many regulatory proteins and protecting cells under stress. Under normal circumstances, HSP90 is non-active and is present at low expression, however when the cells are subjected to various causes of stress, the protection mechanism of HSP90 is activated and HSP90 synthesis increases in cells. On the one hand, HSP90 plays a protective role through signal transduction pathways with the promotion of the appropriate hormone receptor activation or by entering the nucleus, thus displaying its repair activity [42]. In the present study, we found that HSP90 in NC controls is significantly increased. Various stresses lead to liver tissue injury in NC, causing upregulation of HSP90 expression. We postulate that HSP 90 may play a protective role during cholestasis, which may explain the better prognosis observed for NC compared to BA. HSP90 overexpression is anticipated to differentiate the severity in the cholestasis process, especially in the BA and NC, because they have same clinical manifestations. However, further studies are required to verify the current studies and the viability of utilizing HSP90 as a biomarker.

These findings suggest that changes in HSP90 expression may have potential clinical impact. However, the sample size in the current study is relatively small, indicating that the proteomic data need to be confirmed in a study with a greater number of patients and controls. Moreover, more patient-centered and much larger sample numbers are required. However, biomarkers are known to have the potential to dramatically alter options and strategies of diagnosis and treatment, and consequently a complex process with multiple steps of further analyses is necessary for translating our pilot proteomic observations into clinical applications. Future studies are also necessary to determine the role that HSP90 may play in the pathogenesis of BA, which may provide insight into the mechanisms regulating the biliary damage observed in BA.
